# Lactate exposure shapes the metabolic and transcriptomic profile of CD8+ T cells

**DOI:** 10.3389/fimmu.2023.1101433

**Published:** 2023-02-27

**Authors:** Laura Barbieri, Pedro Veliça, Paulo A. Gameiro, Pedro P. Cunha, Iosifina P. Foskolou, Eric Rullman, David Bargiela, Randall S. Johnson, Helene Rundqvist

**Affiliations:** ^1^ Department of Cell and Molecular Biology, Karolinska Institutet, Stockholm, Sweden; ^2^ Department of Physiology, Development and Neuroscience, University of Cambridge, Cambridge, United Kingdom; ^3^ Department of Surgery, Oncology, and Gastroenterology, University of Padova, Padova, Italy; ^4^ RNA Networks Laboratory, Francis Crick Institute, London, United Kingdom; ^5^ Department of Neuromuscular Diseases, University College London, Queen Square Institute of Neurology, London, United Kingdom; ^6^ Department of Laboratory Medicine, Karolinska Institutet, Stockholm, Sweden

**Keywords:** lactate, CD8+ T cells, transcriptome, metabolism, oxygen

## Abstract

**Introduction:**

CD8+ T cells infiltrate virtually every tissue to find and destroy infected or mutated cells. They often traverse varying oxygen levels and nutrient-deprived microenvironments. High glycolytic activity in local tissues can result in significant exposure of cytotoxic T cells to the lactate metabolite. Lactate has been known to act as an immunosuppressor, at least in part due to its association with tissue acidosis.

**Methods:**

To dissect the role of the lactate anion, independently of pH, we performed phenotypical and metabolic assays, high-throughput RNA sequencing, and mass spectrometry, on primary cultures of murine or human CD8+ T cells exposed to high doses of pH-neutral sodium lactate.

**Results:**

The lactate anion is well tolerated by CD8+ T cells in pH neutral conditions. We describe how lactate is taken up by activated CD8+ T cells and can displace glucose as a carbon source. Activation in the presence of sodium lactate significantly alters the CD8+ T cell transcriptome, including the expression key effector differentiation markers such as granzyme B and interferon-gamma.

**Discussion:**

Our studies reveal novel metabolic features of lactate utilization by activated CD8+ T cells, and highlight the importance of lactate in shaping the differentiation and activity of cytotoxic T cells.

## 1 Introduction

Within the adaptive immune system, cytotoxic CD8+ T cells play a central role in controlling viral infections and tumor growth. To accomplish this, T cells are required to rapidly infiltrate various tissues and must therefore be able to adapt to fluctuations in local nutrient levels (1). Upon encountering a cognate antigen, naïve CD8+ T cells undergo a rapid metabolic and transcriptional shift that drives them out of quiescence and into an activated, highly proliferative state (2). This shift is supported by accumulation of transcription factors c-MYC and hypoxia-inducible factor 1 alpha (HIF-1α), leading to increased glucose uptake and utilization *via* the glycolytic pathway (3–5). Enhanced ATP production and increased availability of metabolic intermediates, by-products of the glycolytic flux, allow for rapid expansion and acquisition of an effector phenotype (6).

In addition to the inherent metabolic reprogramming to a glycolytic and anabolic profile, recent scientific advances illustrate that the activation and differentiation of CD8+ T cells are modified by the surrounding metabolic milieu and that the ability to utilize alternative carbon sources can confer a proliferative advantage (7–9).

Lactate is a glycolytic by-product derived from the conversion of pyruvate *via* the lactate dehydrogenase (LDH) enzyme. This allows the regeneration of the NAD+ cofactor, essential for enzyme-catalyzed oxidations (10). Lactate is produced by metabolically active cells and tissues as well as proliferating cells, including activated lymphocytes, and has several functions in the organism, e.g., as energy source, gluconeogenic precursor, and signaling molecule (11–14). A thorough review of lactate sensing and signaling mechanisms can be found here (15). A wide range of mammalian tissues can utilize lactate as a carbon source to fuel their metabolism. In fact, in many cases, lactate is the preferred source of energy (16–18). Inter-cellular exchange of lactate occurs between white and red muscle fibers, skeletal muscle and heart, kidney and liver, as well as between neurons and astrocytes (12, 13), illustrating how lactate can be used in various physiological and pathological circumstances (16, 18–20). Recently, an elegant study from Watson et al. demonstrated the ability of tumor-infiltrating regulatory T cells to adapt their metabolism to utilize lactate, under challenging circumstances (21).

Export of lactic acid is coupled to a proton release which can result in localized acidosis, particularly in poorly vascularized areas and fast-growing tumor masses, or in laboratory *ex vivo* cultures. Healthy tissue microenvironments, however, are efficiently buffered and capable of maintaining a physiologically neutral pH even when large amounts of lactate are produced (22, 23).

There is ample evidence that tumor-derived lactate can affect the functions of different immune cell types including T and NK cells due to intracellular acidification. For instance, lactate-associated acidity, i.e., simultaneous availability of both lactate and H+ in the culture media, has been showed to impair CD8+ T cell motility (24). Interestingly, the authors shown that this effect was specific to CD8+ T cells, as CD4+ T cells were not affected. However, this is one of few studies which carefully distinguished between sodium lactate and lactic acid. Most reports of lactate in the immunological context focus on lactic acid, without considering the distinct role of the lactate anion itself (25–29). Because of the detrimental effects of lactic acid on cell proliferation and, in general, fitness, as a result of these studies lactate has long been regarded as immunosuppressive.

In a recent study, we showed that high intensity exercise increases systemic levels of lactate and alters the metabolism of T cells residing in the spleen and lymph nodes. In addition, daily injections of sodium lactate did not inhibit CD8+ T cell function in mice, they rather enhanced the anti-tumoral properties of CD8+ T cells. Furthermore, *in vitro* exposure to lactate induced the expression of effector markers granzyme B and ICOS in cultured CD8+ T cells (30). In support of this, it was recently shown that lactate - contrary to being immunosuppressive - could support stemness and anti-tumoral function (31) and also rescue CD8+ T-cell cytotoxic function under nutrient deprived conditions (32).

We hypothesize that exposing CD8+ T cells to a pH-neutral environment of high lactate availability during the initial phase of T cell activation will shift cell metabolism towards biosynthesis and enhance the effector profile.

## 2 Materials and methods

### 2.1 Animals

All experiments and protocols were approved by the regional animal ethics committee of Northern Stockholm (dnr N78/15, N101/16). For T cell purification, wild type donor and recipient C57BL/6J (RRID: IMSR_JAX:000664) animals were purchased from Janvier Labs.

### 2.2 Osmolality measurements

Sodium Chloride (Sigma, S5886) and Sodium L-Lactate (Sigma, L7022) solutions were prepared by dissolving each compound into RPMI 1640 (Thermo Fisher, 21875) at a maximum concentration of 500 mM. Solutions were serially diluted, and osmolality measurements were obtained with a micro-osmometer (Löser Messtechnik, D-1000 Berlin 38).

### 2.3 Mouse CD8+ T cell purification, activation and expansion

Spleens were harvested from 8-12 week old C57BL/6J (RRID : IMSR_JAX:000664) mice, mashed over a 40 µm cell strainer (VWR, 10199-654), and CD8+ T cells were purified by positive magnetic bead selection (Miltenyi Biotec, 130-117-044) according to manufacturer’s instructions. Purified CD8+ T cells were counted and cell diameter measured using a Moxi Z mini (Orflo, MXZ001) or a TC20 (Bio-Rad, 145-0101) automated counter. 1x10^6^ (24-well plate) or 5x10^5^ (48-well plate) CD8+ T cells were activated with Dynabeads Mouse T-Activator CD3/CD28 (Thermo Fisher, 11456D) in a 1:1 bead to T cell ratio and cultured in 2 ml (24-well plate) or 1 ml (48-well plate) RPMI 1640 (Thermo Fisher, 21875) supplemented with 10% Fetal Bovine Serum (Thermo Fisher, 10270-106), 50 µM 2-mercaptoethanol (Thermo Fisher, 21985023), 100 U/ml penicillin-streptomycin (Thermo Fisher, 15140122), and 10 U/ml recombinant human IL-2 (Sigma, 11011456001), and incubated at 37˚ C for 3 days in a humidified CO_2_ incubator.

### 2.4 Human CD8+ T cell purification, activation, and expansion

Peripheral blood mononuclear cells (PBMCs) were harvested from standard buffy coat preparations of healthy donors, aged 20 to 40 years old, obtained from the Department of Transfusion Medicine at Karolinska University Hospital and processed no later than 4 hours after collection. PBMCs were isolated by gradient centrifugation using Histopaque-1077 (Sigma, 10771) and enriched for CD8+ T cells by magnetic bead selection, according to manufacturer’s instructions (Miltenyi Biotec, 130-045-201, RRID : AB_2889920). Purified CD8+ T cells were counted and 5x10^5^ (48-well plate) or 1x10^5^ (96-well plate) naïve CD8+ T cells were activated with Dynabeads Human T-Activator CD3/CD28 (Thermo Fisher, 11132D) in a 1:1 bead to T cell ratio and cultured in 1 ml (48-well plate) or 200µl (96-well plate) RPMI 1640 (Thermo Fisher, 21875) supplemented with 10% Fetal Bovine Serum, (Thermo Fisher, 10270-106), 100 U/ml penicillin-streptomycin (Thermo Fisher, 15140122), and 30 U/ml recombinant human IL-2 (Sigma, 11011456001), and incubated at 37˚C for 4 days in a humidified CO_2_ incubator. For long-term expansion after 4 days, Dynabeads were removed using a DynaMag-2 Magnet (Thermo Fisher, 12321D), washed with PBS, counted, and resuspended in fresh media supplemented with 30 U/ml IL-2 and incubated as described above. Media supplemented with cytokines was added every 2-3 days to maintain cell densities below 2x10^6^/ml. Cells were counted again at day 7 and maintained in culture under the above-described conditions until day 14.

### 2.5 CD8+ T cell *ex vivo* activation

Sodium L-Lactate (Sigma, L7022), L-Lactic Acid (Sigma, L1750) and Sodium Chloride (Sigma, S5886) were prepared as 10x concentrated solutions in complete media. Compounds were added to T cells at the point of activation (day 0) minutes before addition of CD3/CD28 Dynabeads. Sodium Chloride and/or plain media was used as control. For long-term expansion, after beads removal cells were cultured in complete media supplemented with cytokines without further addition of compounds.

### 2.6 Cell proliferation analysis

After magnetic bead purification, CD8+ T cells were loaded with 5 µM CellTrace CFSE or CellTrace Violet (CTV) dyes (Thermo Fisher, C34554 and C34557, respectively) according to manufacturer’s instructions. CFSE or CTV dilution was determined by flow cytometry.

### 2.7 Cell phenotyping by flow cytometry

Single-cell suspensions were washed and stained with Fixable Near-IR Dead Cell Stain Kit (Thermo Fisher, L10119) followed by staining of extracellular antigens with fluorochrome labelled antibodies. The Fixation/Permeabilization Solution Kit (BD Biosciences, 554714) was used for exposing cytoplasmic antigens. The Transcription Factor Buffer Set (BD Biosciences, 562574, RRID : AB_2869424) was used for exposing nuclear antigens. Fluorochrome-labelled antibodies against mouse antigens CD44 (clone IM7), CD8 (clone 53- 6.7), CTLA-4 (clone UC10-4F10-11), LAG-3 (clone C9B7W), and PD-1 (clone J43) were purchased from BD Biosciences, CD27 (clone LG.3A10), CD28 (clone 37.51), CD62L (clone MEL-14), and ICOS (clone C398.4A) were purchased from BioLegend, and 4-1BB (clone 17B5), CD127 (clone A7R34), CD25 (clone PC61.5), Eomes (clone Dan11mag), T-bet (clone eBio4B10), and anti-human/mouse Granzyme B (clone GB12) was purchased from Thermo Fisher. Cell counting was performed with CountBright Absolute Counting Beads (Thermo Fisher, C36950). Samples were processed in a FACS Canto II flow cytometer (BD Biosciences). Data analysis was performed with FlowJo (RRID : SCR_008520, version 10.7.2).

### 2.8 Western blotting and real time RT-PCR

Mouse CD8+ T cells were activated *ex vivo* and cells harvested at 0, 6, 12, 24, 48, and 72 hours post-activation. For each time point, total protein was extracted using the NE-PER™ Nuclear and Cytoplasmic Extraction Reagents kit (78833, Thermo Fisher) and the cytoplasmic fraction used for western blots. Total RNA was extracted using the RNeasy Mini Kit (74104, Qiagen). For each sample, 15 µg of total protein were separated in SDS-PAGE, blotted onto a PVDF membrane and probed with antibodies against MCT1 (Santa Cruz Biotechnology Cat# sc-50325, RRID : AB_2083632), MCT4 (Santa Cruz Biotechnology Cat# sc-50329, RRID : AB_2189333), GzmB (Cell Signaling Technology Cat# 4275, RRID : AB_2114432) and PPIB (ABclonal Cat# A7713, RRID : AB_2771764) and detected using infra-red labeled secondary antibodies in an Odyssey imaging system (LICOR). Western blot data were analyzed with the Image Studio Lite software (Image Studio Lite, RRID : SCR_013715). REVERT Total protein stain (926-11010, Li-COR) was used for lane normalization. Two micrograms of total RNA were reverse transcribed using iScript cDNA synthesis kit (BioRad) in a total volume of 20 μl. Real-time RT-PCR was used for mRNA quantification (7500 Fast Real-Time PCR system, Applied Biosystems Inc., Foster City, California, USA). All primers were designed to cover exon-exon boundaries to avoid amplification of genomic DNA. Predesigned qPCR primers used to detect mouse Slc16a1 (forward: 5’-ACTTGCCAATCATAGTCAGAGC-3’, reverse: 5’-CGCAGCTTCTTTCTGTAACAC-3’), Slc16a3 (forward: 5’-GACGCTTGTTGAAGTATCGATTG-3’, reverse: 5’-GCATTATCCAGATCTACCTCACC-3’), granzyme B (GzmB) (forward: 5’-CTGCTAAAGCTGAAGAGTAAG-3’, reverse: 5’-TAGCGTGTTTGAGTATTTGC-3’), and Hprt (forward: 5’-TGACACTGGCAAAACAATGCA-3’, reverse: 5’-GGTCCTTTTCACCAGCAAGCT-3’) were purchased from IdtDNA or Sigma. All reactions were performed in 96-well MicroAmp Optical plates in duplicates. The total reaction volume was 15 µl, containing 5 µl sample cDNA, 0.4 mM of each primer forward and SYBR Green PCR Master Mix (4309155E, Applied Biosystems Ins.). All quantification reactions were controlled with a melting curve and primer efficiency was tested with standard curves, and did not differ between the primer pairs. Gene expression data was normalized to Hprt levels.

### 2.9 RNA sequencing

Mouse CD8+ T cells were activated for 72 hours in the presence of either 40 mM sodium lactate or plain media. At the end of activation cells were washed twice with PBS and cell pellets were snap-frozen in RLT Plus lysis buffer (Qiagen, #1053393). Total RNA was extracted with the Qiagen’s RNeasy kit according to the manufacturer’s instructions. All samples were quality checked with Agilent Tapestation RNA screen tape. To construct libraries suitable for Illumina sequencing, the Illumina TruSeq Stranded mRNA Sample preparation protocol which includes cDNA synthesis, ligation of adapters, and amplification of indexed libraries was used. The yield and quality of the amplified libraries were analyzed using Qubit by Thermo Fisher and the Agilent Tapestation. The indexed cDNA libraries were normalized and combined, and the pools were sequenced on the Nextseq 550 (Illumina NextSeq 550 System, RRID : SCR_016381) for a 50-cycle v2.5 sequencing run, generating 71 bp single-end reads (and 2*10 bp index reads). Demultiplexing was performed using bcl2fastq (bcl2fastq, RRID : SCR_015058, v2.20.0.422), generating fastq files for further downstream mapping and analysis.

### 2.10 RNA sequencing analysis

Bcl files were converted and demultiplexed to fastq using the bcl2fastq v2.20.0.422 program. STAR 2.7.5b (33) was used to map the fastq files to the mouse reference genome (mm10/GRCm38) and to remove PCR duplicates. Uniquely mapped reads were then counted in annotated exons using featureCounts v1.5.1 (34). The gene annotations (Mus_musculus.GRCm38.99.gtf) and reference genome were obtained from Ensembl. The count table from featureCounts was imported into R/Bioconductor and differential gene expression was performed using the EdgeR package (35) and its general linear models pipeline. For the gene expression analysis, genes that had 1 count per million in 3 or more samples were used and normalized using TMM normalization. Genes with an FDR adjusted p value ≤ 0.01 were termed significantly regulated. Principal Component Analysis (PCA) plots were obtained by normalizing gene counts after filtering out lowly expressed genes.

### 2.11 Metabolite extraction

For metabolite analysis, 5x10^6^ cells were harvested and washed 3 times with PBS before adding 800 µL of ice-cold extraction mixture consisting of chloroform:MeOH:H2O (1:3:1), containing 500 pg/µL of 2-Deoxy-D-glucose 6-phosphate and 70 pg/µL of myristic acid-^13^C_3_ were added to each sample. Metabolites were extracted using a mixer mill set to a frequency 30 Hz for 2 min, with 1 tungsten carbide bead added to each tube. Obtained extracts were centrifuged at 14000 rpm for 10 min. The collected supernatants were divided for GC-MS and Sugar-P analysis. 300 µL of the supernatant was transferred into GC and LC-vial respectively, the supernatants were evaporated until dryness using a SpeedVac.

### 2.12 Sugar phosphate analysis

For derivatization, dried samples were dissolved in 20 µl of methoxylamine and incubated on a heat block at 60°C, 30 min. After overnight incubation at room temperature, 12µl of 1- Methylimidazol and 6 µl of propionic acid anhydride were added and heated at 37°C for 30 minutes. The reaction mixture was then evaporated to dryness by N2 gas. Prior to LC-MS analysis, derivatized metabolites were dissolved in 100µl of aqueous 0.1% formic acid. Quantitative analysis was performed by combined ultra-high-performance liquid chromatography electrospray ionization-triple quadrupole-tandem mass spectrometry (UHPLC-ESI-QqQMS/MS) in dynamic multiple-reaction-monitoring (MRM) mode. An Agilent 6495 UHPLC chromatograph equipped with a Waters Acquity BEH 1.7 µm, 2.1 x 100 mm column (Waters Corporation, Milford, USA) coupled to a QqQ-MS/MS (Agilent Technologies, Atlanta, GA, USA) was used. The washing solution, for the autosampler syringe and injection needle, was 90% MeOH with 1% HCOOH. The mobile phase consisted of A, 2% HCOOH and B, MeOH with 2% HCOOH. The gradient was 0% B for 1 min followed by linear gradients from 0.1 to 30% from 1 to 3 min then 30 to 40% B from 3 to 6 min, hold at 40% B from 6 to 10 min, followed by 40 to 70% B from 10 to 12.5 min, hold at 70% B from 12.5 to 15 min, and thereafter 70 to 99% B from 15 to 17.5 min. B was held at 99% for 0.5 min, and thereafter the column was re-equilibrated to 0% B. The flow rate was 0.65 mL min-1 during equilibration and 0.5 mL min-1 during the chromatographic runs. The column was heated to 40°C, and injection volumes were 1 μL. The mass spectrometer was operated in negative ESI mode with gas temperature 230°C; gas flow 12 L min-1; nebulizer pressure 20 psi; sheath gas temperature 400°C; sheath gas flow 12 L min-1; capillary voltage 4000 V (neg); nozzle voltage 500 V; iFunnel high pressure RF 150 V; iFunnel low pressure RF 60 V. Fragmentor voltage 380 V and cell acceleration voltage 5 V. Data were processed using MassHunter Qualitative Analysis and Quantitative Analysis (QqQ; Agilent MassHunter WorkStation - Qualitative Analysis for GC/MS, RRID : SCR_016657) and Excel (Microsoft, Redmond, Washington, USA) software.

### 2.13 GC-MS analysis

The GC-MS samples were spiked with 1050 pg of each GC-MS internal standard before evaporation. Derivatization was performed according to Gullberg et al. (36). In detail, 10µL of methoxyamine (15 µg/µL in pyridine) was added to the dry sample that was shaken vigorously for 10 minutes before being left to react at room temperature. After 16 hours 10 µL of MSTFA was added, the sample was shaken and left to react for 1 hour at room temperature. 10 µL of methyl stearate (1050 pg/µL in heptane) was added before analysis. One µL of the derivatized sample was injected by an Agilent 7693 autosampler, in splitless mode into an Agilent 7890A gas chromatograph equipped with a multimode inlet (MMI) and 10 m x 0.18 mm fused silica capillary column with a chemically bonded 0.18 μm DB 5-MS UI stationary phase (J&W Scientific). The injector temperature was 260°C. The carrier gas flow rate through the column was 1 ml min-1, the column temperature was held at 70°C for 2 minutes, then increased by 40°C min-1 to 320°C and held there for 2 min. The column effluent 16 is introduced into the electron impact (EI) ion source of an Agilent 7000C QQQ mass spectrometer. The thermal AUX 2 (transfer line) and the ion source temperatures were 250°C and 230°C, respectively. Ions were generated by a 70 eV electron beam at an emission current of 35 µA and analyzed in dMRM-mode. The solvent delay was set to 2 minutes. Data were processed using MassHunter Qualitative Analysis and Quantitative Analysis (QqQ; Agilent Technologies, Atlanta, GA, USA) and Excel (Microsoft, Redmond, Washington, USA) software.

### 2.14 Isotopic labelling, metabolite extraction and GC-MS analysis

Mouse CD8+ T cells were activated with CD3/CD28 beads and 10 U/ml IL-2 in glucose-free RPMI supplemented with 2 mM glutamine, 10% FBS, 100 U/ml penicillin/streptomycin and 2-mercaptoethanol, and either i) 11 mM [U^13^C_6_] glucose, ii) 11mM [U^13^C_6_] glucose and 40 mM lactate, or iii) 11 mM glucose and 40 mM [U^13^C_3_] sodium lactate, and isotopic labelling was performed for 24, 48 and 72 hours. Sodium chloride (40 mM) was added to i) to control for osmolality. Cells harvested at each time point were washed with PBS, and metabolic activity quenched by freezing samples in dry ice and ethanol, and stored at -80°C. Metabolites were extracted by addition of 600 μl ice-cold 1:1 (vol/vol) methanol/water (containing 1 nmol scyllo-Inositol as internal standard) to the cell pellets, samples were transferred to a chilled microcentrifuge tube containing 300 μl chloroform and 600 μl methanol (1500 μl total, in 3:1:1 vol/vol methanol/water/chloroform). Samples were sonicated in a water bath for 8 min at 4°C, and centrifuged (13000 rpm) for 10 min at 4°C. The supernatant containing the extract was transferred to a new tube for evaporation in a speed-vacuum centrifuge, resuspended in 3:3:1 (vol/vol/vol) methanol/water/chloroform (350 μl total) to phase separate polar metabolites (upper aqueous phase) from apolar metabolites (lower organic phase), and centrifuged. The aqueous phase was transferred to a new tube for evaporation in a speed-vacuum centrifuge, washed with 60 μl methanol, dried again, and derivatized by methoximation (20 μl of 20 mg/ml methoxyamine in pyridine, RT overnight) and trimethylsilylation (20 μl of N,O-bis(trimetylsilyl)trifluoroacetamide + 1% trimethylchlorosilane) (Sigma, 33148) for ≥ 1 h. GC-MS analysis was performed using an Agilent 7890B-5977A system equipped with a 30 m + 10 m × 0.25 mm DB-5MS + DG column (Agilent J&W) connected to an MS operating in electron-impact ionization (EI) mode. One microliter was injected in splitless mode at 270°C, with a helium carrier gas. The GC oven temperature was held at 70°C for 2 min and subsequently increased to 295°C at 12.5°C/min, then to 320°C at 25°C/min (held for 3 min). MassHunter Workstation (B.06.00 SP01, Agilent Technologies) was used for metabolite identification by comparison of retention times, mass spectra and responses of known amounts of authentic standards. Metabolite abundance and mass isotopologue distributions (MID) with correction of natural 13C abundance were determined by integrating the appropriate ion fragments using the GC–MS Assignment Validator and Integrator (GAVIN) (37).

### 2.15 Quantification of interferon-gamma secretion

Soluble Interferon-gamma (IFN-γ) was quantified in culture media 3 days after CD8+ T cell activation using the Mouse IFN-gamma Quantikine ELISA Kit (RnD Systems, MIF00).

### 2.16 Oxygen consumption rate and extracellular acidification rate measurements

CD8+ T cells activated for 3 days were washed and oxygen consumption rate (OCR) and extracellular acidification rate (ECAR) in a Seahorse Extracellular Flux Analyzer XF96 (Agilent). 3x10^5^ CD8+ T cells were plated onto poly-D-lysine coated wells and assayed in XF RPMI medium (Agilent) pH 7.4 supplemented with 10 mM glucose and 2 mM glutamine. A minimum of 4 technical replicates per biological replicate was used. During the assay, wells were sequentially injected with 1 µM oligomycin (Sigma), 1.5 µM FCCP (Sigma) and 100 nM rotenone (Sigma) + 1 µM antimycin A (Sigma).

### 2.17 Glycolytic stress test of CD8+ T cells 12 hours after activation

Purified CD8+ T cells were activated for 12 hours, then 3x10^5^ CD8+ T cells were plated onto poly-D-lysine coated wells in order to form a homogeneous monolayer. Cells were assayed in XF RPMI medium (Agilent) pH 7.4 supplemented with 2 mM glutamine. A minimum of 4 technical replicates per each biological replicate was used. Oxygen consumption rate (OCR) and extracellular acidification rate (ECAR) were determined through a Seahorse Extracellular Flux Analyzer XF96 (Agilent). During the assay, wells were sequentially injected with 40 mM NaCl/NaLac with or without the MCT1-selective inhibitor AZD3965 (Cayman Chemical, 19912) or the LDH inhibitor GSK2837808A (Bio-Techne, 5189), or plain media alone, followed by 10 mM glucose (Sigma), 1 µM oligomycin and 50 mM 2-deoxyglucose (2-DG; Sigma D6134).

### 2.18 Transcription factor analysis

Analysis of transcription factor regulators was carried out on transcripts abundantly expressed (1^st^ quartile) and up-regulated with lactate exposure (n = 685) queried against positional weight matrixes of human and rodent transcription factors from Transfac and JASPER. EnrichR (38), combined with single-cell sequencing data from human T-cells (39) were used. Transcription factors detected at the transcriptional levels in human T cells and in the present experiment with a motif-enrichment FDR of <5% were deemed as transcriptional regulators.

### 2.19 Statistics

Statistical analyses were performed with Prism 9 version 9.2.0 (GraphPad). Statistical tests and number of replicates are stated in figure legends.

### 2.20 Study approval

All animal experiments were approved by the regional animal ethics Committee of Northern Stockholm, Sweden (dnr N78/15 and N101/16). The Stockholm Regional Ethical Review Board does not require an ethical permission for the use of non-identified healthy donor blood samples.

## 3 Results

### 3.1 Exposure to sodium lactate during activation is well tolerated by CD8+ T cells and alters the expression of effector molecules towards a more cytotoxic profile

**Figure 1:**
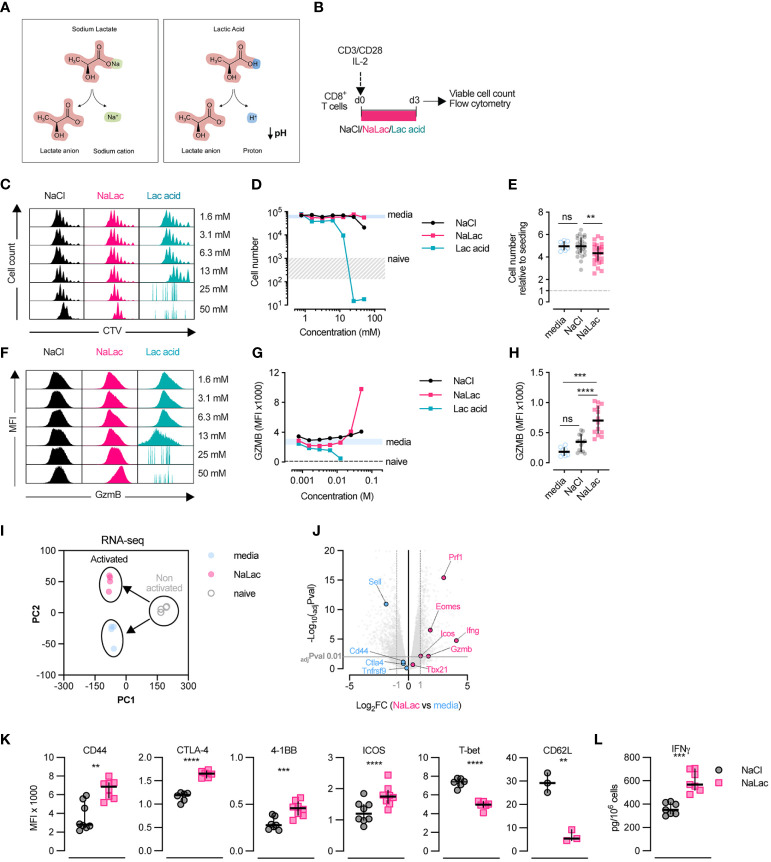
Sodium lactate, but not lactic acid, is well tolerated by CD8+ T cells. **(A)** Diagram representing the dissociation of sodium lactate (NaLac, left) and lactic acid (Lac acid, right) in aqueous solution. **(B)** Experimental design. Mouse CD8+ T cells were activated for 72 hours in the presence of varying concentrations of sodium chloride (NaCl), sodium lactate (NaLac), or lactic acid (Lac acid). After activation, culture media was removed, cells were washed, counted, and analyzed by flow cytometry. **(C)** Flow cytometry histograms from experiment in panel **(B)**, showing cellular division profile by means of CellTrace Violet (CTV) dilution. **(D)** Effect of increasing concentrations of NaCl, NaLac, and lactic acid on CD8+ T cell number, 72 hours after activation. Grey area: range of cell number for naive (non-activated) cells; Solid blue line: average of non-treated (culture media) CD8+ T cells. N = 1 composed of two mouse spleens pooled together. **(E)** Number of mouse CD8+ T cells, 72 hours after activation in the presence of 40 mM NaCl, or NaLac, or plain culture media. Data are the median and interquartile range of n = 26 biological replicates. Cell number is presented relative to the number of cells seeded (dotted line). **P<0.001 Dunnett’s multiple comparisons test. **(F)** Flow cytometry histograms showing the median fluorescence intensity (MFI) of Intracellular granzyme B (GZMB) at each cell division of mouse CD8+ T cells activated as in **(B)**, determined by CTV peaks. **(G)** Median fluorescence intensity (MFI) of GZMB levels at increasing doses of the different treatments at each cell division. Solid light blue line: range (minimum to maximum) of media-treated, activated CD8+ T cells (control); dotted line: average of non-activated CD8+ T cells (naïve). **(H)** Median fluorescence intensity (MFI) of GZMB measured by flow cytometry, at day 3 after activation with 40 mM NaCl, 40 mM of NaLac, or plain media for 72 hours (same experiment as in **(E)**). Data are the median and interquartile range of n = 17 biological replicates. ****P<0.0001 Dunnett’s multiple comparisons test. ns, non-significant **(I)** Principal Component Analysis (PCA) plot of RNA-seq data from mouse CD8+ T activated with either 40 mM sodium lactate (NaLac, pink), or media control (light blue), or non-activated (naïve, grey). Each dot represents a sample, while each color represents a treatment. RNA was purified from 4 independent mouse donors per condition. **(J)** Volcano plot of the RNA-seq data, showing genes associated with T cell differentiation and function that are differentially expressed in NaLac- versus media-treated, activated CD8+ T cells. Significantly up- or down-regulated genes (Log2 fold change ≥ 1 or ≤ -1) are highlighted in pink or light blue, respectively. P values are calculated from Student’s t-test and adjusted by false discovery rate (FDR). Significance is set at adjusted p value (adjPval) ≤ 0.01. Grey horizontal line indicates adjPval = 0.01. Dashed vertical lines indicate the threshold of differentially expressed transcripts (Log2 fold change ≥ 1 and ≤ -1). **(K)** Median fluorescence intensity (MFI) of CD44, CTLA-4, 4-1BB, ICOS, T-bet and CD62L analyzed by flow cytometry, in mouse CD8+ T cells activated for 72h in the presence of 40 mM NaCl or 40 mM NaLac. Each pair represents an independent mouse donor (n = 3-9). **P<0.01, ***P<0.001, ****p<0.0001 two-tailed paired t-test. **(L)** Levels of interferon-γ (IFN-γ) secreted by mouse CD8+ T cells activated as in **(K)**.

In metabolically perturbed tissues, glycolysis will lead to protonated lactate, i.e., lactic acid, being released into the extracellular environment. The subsequent dissociation of the lactate anion from the proton leads to a localized decrease in pH (**Figure 1A**). In the physiological setting, a reduction in pH is counteracted by ample buffering systems, such as the bicarbonate buffer (22). In order to test the effect of lactate on CD8+ T cell activation and differentiation, independently of pH reduction, we activated purified mouse CD8+ T cells *ex vivo* for 3 days in the presence of increasing doses of either lactic acid, or its pH-neutral salt sodium lactate, or sodium chloride as a control for osmolality (**Figure 1B** and **Supplementary Figure 1A**). This allowed us to distinguish between the effects of the lactate anion itself and the lactate anion in conjunction with acidification. Addition of lactic acid to culture media caused a sharp decrease in pH, while equimolar doses of sodium lactate or sodium chloride did not alter media pH (**Supplementary Figure 1B**). As reported previously (40), pH-lowering lactic acid has deleterious effects on CD8+ T cell activation, resulting in significantly reduced proliferation and cell numbers (**Figures 1C, D**). In contrast, exposure to increasing doses of pH-neutral sodium lactate, for 72 hours during TCR triggering, did not impair cell expansion and had only a minimal effect on cell proliferation (**Figures 1C–E** and **Supplementary Figure 1C**). A decrease in proliferation was observed at NaCl and sodium lactate concentrations above 50 mM, likely due to increased osmolality (**Figures 1C, D**). Based on this, we selected 40 mM as the highest tolerated dose of sodium lactate that could induce a significant effect without irreversibly impairing cell fitness (**Figure 1E**). Interestingly, human CD8+ T cells activated in the presence of increasing doses of sodium lactate or sodium chloride showed a higher sensitivity to the rise in osmolality, but overall, a similar response to the one of murine cells (**Supplementary Figure 1D**). Exposure to 40 mM sodium lactate during activation was well tolerated by human CD8+ T cells, with no significant impact over prolonged *in vitro* culture (**Supplementary Figure 1E**).

In line with our findings from a previous publication (30), addition of sodium lactate during activation elicited a dose-dependent increase in Granzyme B (GZMB) expression, beginning at 10 mM and peaking at 40-50 mM (**Figures 1F–H** and **Supplementary Figures 1F, G**). Granzyme B is one of the most highly expressed effector proteins in activated mouse CD8+ T cells (41) and essential to granule-mediated apoptosis of target cells (42), suggesting that lactate can alter the effector profile of CD8+ T cells.

Aiming to expand our understanding of the role of lactate during T cell activation, we compared the transcriptome of CD8+ T cells prior activation (naïve) and 72 hours after activation in the presence of media or 40 mM sodium lactate. TCR triggering caused a drastic reprogramming of the cellular transcriptome, which was further altered by the presence of lactate (**Figure 1I** and **Supplementary Figures 1H, I**). Specifically, lactate had a substantial effect on the expression of genes involved in CD8+ T cell differentiation and cytotoxic activity, including Granzyme B (Gzmb), interferon-gamma (IFN-γ) (Ifng), perforin 1 (Prf1) and ICOS (Icos) (**Figure 1J**). By means of flow cytometry, we could confirm that lactate also altered protein levels of a range of factors involved in CD8+ T cell differentiation, increasing the levels of CD44, CTLA-4, 4-1BB, and ICOS, and lowering the levels of the transcription factor T-bet and the lymph node homing molecule CD62L (**Figure 1K** and **Supplementary Figure 1I**). In addition, CD8+ T cells activated with 40 mM sodium lactate showed increased secretion of IFN-γ, a key mediator of cytotoxicity (**Figure 1L**).

### 3.2 Exogenous lactate acutely suppresses glycolysis and promotes oxidative metabolism in activated CD8+ T cells

**Figure 2:**
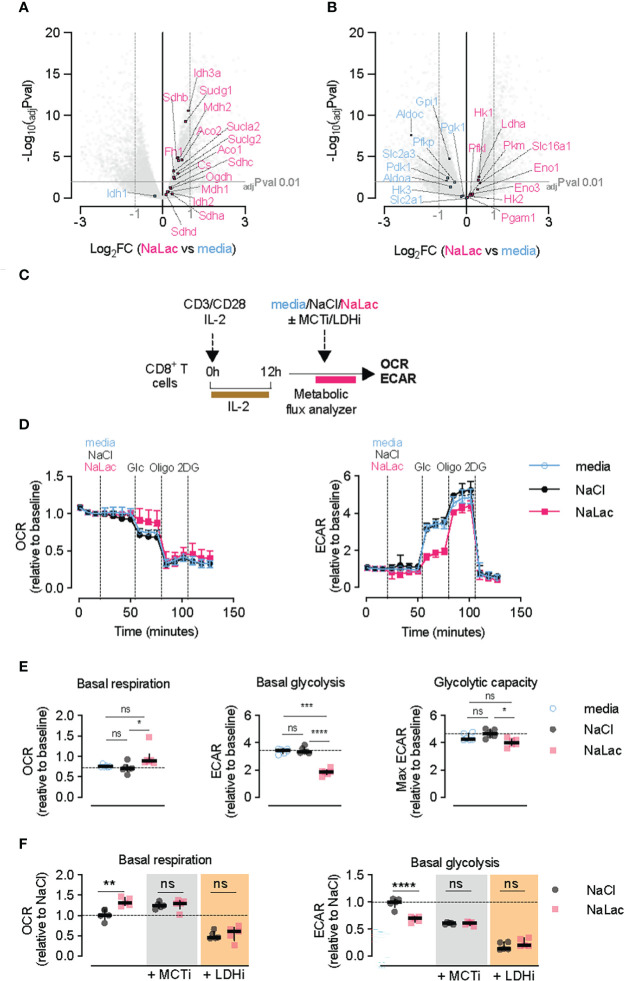
Lactate modulates CD8+ T cell metabolism. **(A, B)** Volcano plot generated from the RNA-seq data, representing differentially expressed TCA cycle **(A)** or glycolytic **(B)** genes in NaLac- relative to media-treated CD8+ T cells, 72 hours after activation. Significantly up- or down-regulated genes are highlighted respectively in pink or light blue. P values were calculated using Student’s t-test and adjusted by false discovery rate (FDR). Significance was set at adjusted p value (adjPval) ≤ 0.01, as indicated by the grey horizontal line. Dashed vertical lines indicate the threshold of differentially expressed transcripts (Log2 fold change ≥ 1 and ≤ -1). **(C)** Experimental design. CD8+ T cells were purified from mouse splenocytes and activated with anti-CD3/CD28 beads and IL-2 in media. After 12 hours, cells were counted, transferred into a metabolic flux analyzer, and exposed to either 40 mM NaCl, 40 mM sodium lactate (NaLac), or culture media prior to undergoing a glycolytic stress test. **(D)** Glycolytic stress test of mouse CD8+ T cells 12 hours after activation. Extracellular acidification rate (ECAR) and oxygen consumption rate (OCR) during injection of either media, 40 mM NaCl, or 40 mM NaLac, followed by 10 mM glucose, 1 µM oligomycin (oligo) and 50 mM 2-deoxyglucose (2-DG). ECAR and OCR are normalized to baseline levels. Data are the median and interquartile range of n = 6 independent mice. **(E)** Basal mitochondrial respiration, basal glycolysis and glycolytic capacity determined from the assay shown in **(D)**. Dotted line: median of NaCl-treated cells. Data is the median and interquartile range of n = 6 independent mouse donors, each assayed as 4 technical replicates. *P<0.01, ***P<0.0005, ****p<0.0001 RM one-way ANOVA with Tukey’s multiple comparisons test. **(F)** Basal mitochondrial respiration and basal glycolysis determined from cells treated as in **(C)**, and after injection of either NaCl or NaLac with or without addition of 10 nM of the monocarboxylate transporter-1 (MCT-1) inhibitor AZD3985 or 50 μM of the lactate dehydrogenase (LDH) inhibitor GSK2837808A. Data are the median and interquartile range of n = 5 independent mouse donors. **P<0.001, ****p<0.0001 two-way ANOVA with Šídák’s multiple comparisons test. ns, non significant.

In addition to altering the expression of effector molecules, further analysis of the transcriptome revealed that exposure to sodium lactate during the activation window induced a concerted upregulation of genes involved in the TCA cycle (**Figure 2A** and **Supplementary Figure 2A**). In contrast, expression of genes involved in glycolysis was not uniformly affected by lactate compared to media alone (**Figure 2B** and **Supplementary Figure 2A**).

The significant shift in metabolic gene expression led us to characterize the immediate effects of lactate exposure on CD8+ T cell metabolism. For this purpose, a short activation signal was given 12 hours before carrying out real-time metabolic analyses. During the metabolic flux monitoring, 40 mM of sodium lactate was administered to the cells (**Figure 2C**). Relative to cells treated with media or an equimolar dose of NaCl, addition of sodium lactate reduced the extracellular acidification rate (ECAR) of activated CD8+ T cells and concomitantly increased the oxygen consumption rate (OCR), used as surrogate measurements for glycolysis and oxidative respiration, respectively (**Figures 2D, E**). Despite suppressing glycolysis, addition of sodium lactate only slightly reduced the glycolytic capacity of the cells (**Figures 2D, E**). Similar effects were observed in human CD8+ T cell cultures (**Supplementary Figures 2B–D**), suggesting that exogenous lactate is taken up by metabolically active CD8+ T cells and instantly shifts down the endogenous production of lactic acid in the activated cells.

As previously described, upon TCR triggering, CD8+ T cells undergo a rapid induction of glycolytic metabolism which is followed by an increase in both intracellular and extracellular lactate levels (**Supplementary Figures 2E, F**). The lactate anion is transported across cell membranes *via* the proton-linked monocarboxylate transporters (MCT1, 2, 3, and 4) (43, 44). Of these, only MCT1 and MCT4 are expressed in CD8+ T cells (41, 45, 46). While neither transporter is expressed at high levels in naïve CD8+ T cells, *ex vivo* activation causes MCT1 mRNA and protein to peak at 12 to 24 hours post-activation, whereas MCT4 expression peaks later, between 48 and 72 hours after activation (**Supplementary Figures 2G, H**). Activation of CD8+ T cells in the presence of an MCT1-selective inhibitor (AZD3965) inhibited cell proliferation at the low nanomolar range, irrespective of the presence of lactate (**Supplementary Figure 2I**), suggesting a crucial role for monocarboxylate transport in early T cell expansion.

To confirm that the metabolic effect observed in **Figures 2C–E** was due to the presence of exogenous lactate, we assessed the glycolytic and oxidative rates of T cells after administration of lactate in combination with an MCT1 inhibitor (**Figure 2F**). Blocking the membrane trafficking of lactate *via* MCT1 mimicked the effects of sodium lactate, causing reduced ECAR and increased OCR (**Figure 2F**). This suggests that retention of endogenously generated lactate impacts T cell metabolism in a similar way as increased exogenous lactate. As shown in **Figure 2F**, adding exogenous sodium lactate during treatment with the MCT1 inhibitor did not further alter the T cell metabolic parameters, supporting efficient blocking of lactate transport and a dependence on an active uptake for the metabolic effects seen with lactate.

The lactate dehydrogenase enzyme (LDH) plays a crucial role in lactate metabolism as it catalyzes the interconversion between pyruvate and lactate. Activated CD8+ T cells express high levels of LDHA and lower levels of LDHB (**Supplementary Figure 2J**) (47). We hypothesized that inhibition of LDH would prevent incorporation of exogenous lactate into carbon metabolism and abrogate its effects. However, inhibition of LDH activity with the LDH inhibitor GSK2837808A (48) caused a drastic reduction of T cell expansion (**Supplementary Figure 2K**), and led to suppression of both basal glycolytic and oxidative respiration rates, in both the presence and absence of exogenous lactate (**Figure 2F**). This result highlights the central role played by LDH in the metabolism of activated CD8+ T cells, and indicates that disrupting the shuttling between pyruvate and lactate has dramatic consequences for the metabolic fitness of the cell. However, due to the deleterious effects on cell viability, inhibition of LDH did not allow us to determine if the effects of exogenous lactate on CD8+ T cell metabolism are directly dependent on the LDH-mediated conversion to pyruvate.

### 3.3 Prolonged exposure to lactate alters the CD8+ T cell metabolome

**Figure 3:**
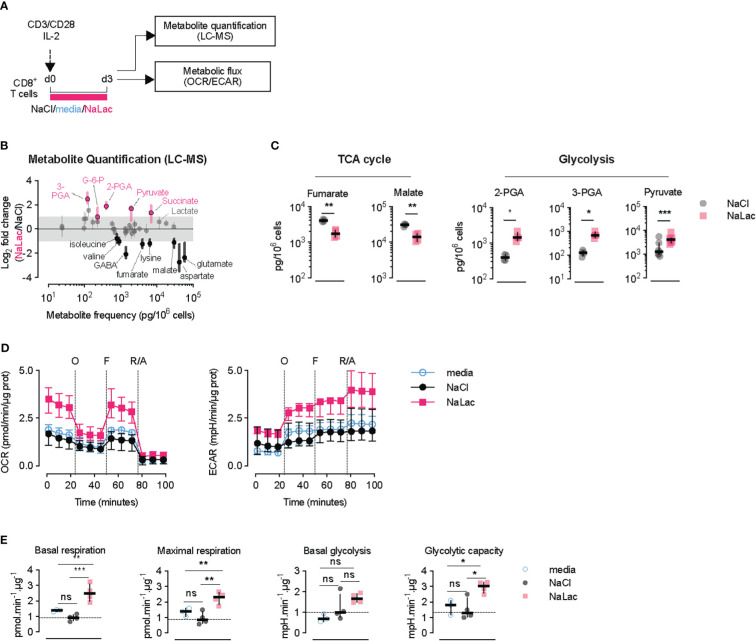
Early exposure to lactate alters CD8+ T cell metabolism and gene expression. **(A)** Experimental design. CD8+ T cells were purified from mouse splenocytes and activated with anti-CD3/CD28 beads and IL-2 in the presence of either plain media, or 40 mM NaCl, or 40 mM NaLac for 72 hours. Cells were then analyzed by liquid chromatography-mass spectrometry (LC-MS) and metabolic flux analysis (OCR/ECAR). **(B)** Metabolic changes after 72 hours-activation of mouse CD8+ T cells in the presence of NaLac compared to NaCl. Metabolites were extracted and quantified by Sugar Phosphate and Gas Chromatography-Mass Spectrometry (GC-MS). Pyruvate levels were determined by colorimetric assay. Data show log2 fold changes between NaLac- and NaCl-treated samples of n = 4 independent mouse donors. Pink and black dots represent significantly increased or decreased metabolite frequency in NaLac- versus NaCl-treated cells, respectively. Grey area: range between 2-fold increase and 2-fold decrease. **(C)** Paired analysis of significant metabolites from the same assay as in **(D)**, grouped by metabolic pathway (n = 4, with each pair representing an independent mouse donor). Pyruvate levels were determined by a colorimetric assay from n = 7 independent mouse donors. **P<0.01, *P<0.05 two-tailed paired t test. **(D)** Oxygen consumption rate (OCR) and extracellular acidification rate (ECAR) of CD8+ T cells after 72 hours activation in the presence of 40 mM NaCl, 40 mM NaLac, or plain media. OCR and ECAR were measured during sequential addition of 1 µM oligomycin (oligo), 1.5 µM FCCP, and 100 nM rotenone combined with 1 µM antimycin A (Rot/Ant). OCR and ECAR were normalized to total protein content at the end of assay. Lines represent the median and interquartile range of n = 4 independent mouse donors, each assayed as 4 technical replicates. **(E)** Basal respiration, maximal respiration, basal glycolysis, and glycolytic capacity determined from the assay shown in **(F)**. Dotted line: median of NaCl-treated (control) cells. *P<0.05, **P<0.001, ***P<0.001 one-way ANOVA with Tukey’s multiple comparison test. ns, non significant.

To understand how T cell metabolism is altered by prolonged exposure to lactate during TCR triggering, metabolite quantification by mass spectrometry and metabolic flux analysis was conducted after activation of CD8+ T cells for 72 hours in the presence of 40 mM sodium lactate (**Figure 3A**). Mass spectrometric analysis of the T cell metabolome revealed increased levels of glycolytic intermediates, including glucose-6-phosphate (G-6-P), 3- and 2-phosphoglyceric acid (3-PGA, 2-PGA) and pyruvate, in CD8+ T cells exposed to sodium lactate for 3 days (**Figures 3B, C** and **Supplementary Figure 3A**). Within the TCA cycle intermediates, lactate exposure caused an increase in the amount of succinate and a reduction in the downstream metabolites fumarate and malate (**Figures 3B, C** and **Supplementary Figure 3A**). Mitochondrial stress tests confirmed that CD8+ T cells activated in the presence of sodium lactate for 72 hours exhibit higher basal and maximal respiration as well as increased glycolytic rates and glycolytic capacity at the end of the exposure (**Figures 3D, E**). Altogether, these results demonstrate that CD8+ T cells adapt with an enhanced metabolic capacity when exposed to lactate during activation, by increasing the flux through glycolysis and the TCA cycle. The increased levels of pyruvate after lactate exposure and the shift in TCA metabolites indicate that lactate is taken up and incorporated into cellular metabolism.

### 3.4 Lactate is incorporated in cellular metabolism and displaces glucose for TCA derived metabolites and amino acids

**Figure 4:**
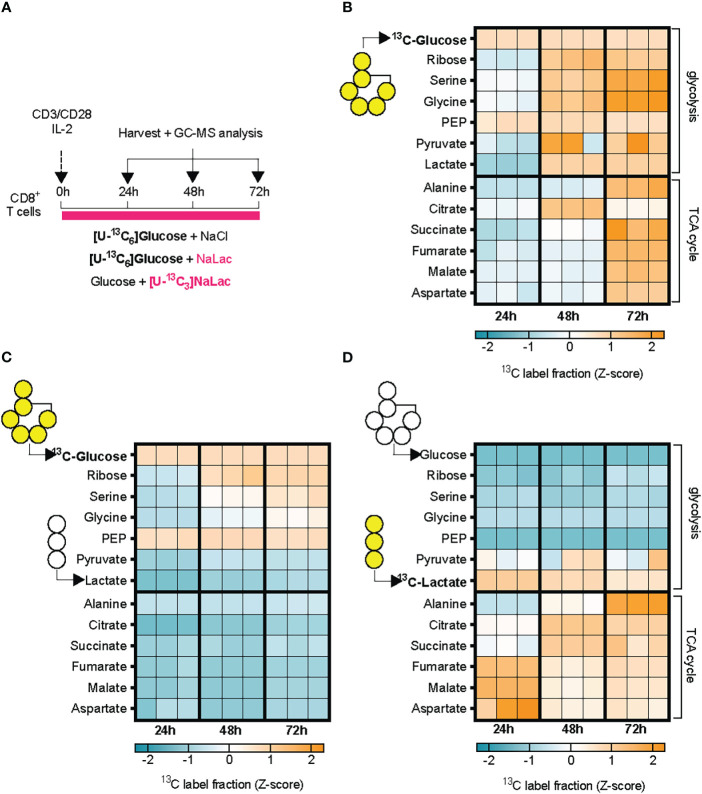
Lactate is incorporated in cellular metabolism and displaces glucose. **(A)** Experimental design. Mouse CD8+ T cells were activated in the presence of either 11.1 mM [U-13C6]glucose + 40 mM NaCl, or 11.1 mM [U-13C6]glucose +40 mM sodium lactate, or 11.1 mM glucose and 40 mM [U-13C6]sodium lactate. Incorporation of labelled carbons in cellular metabolites was assessed by GC-MS at 24, 48, and 72 hours after activation. **(B–D)** Heatmaps showing glucose- **(B, C)** or lactate-derived **(D)** carbons incorporation into cellular metabolites at 24, 48, and 72 hours after activation, expressed as Z-scores. N = 3 individual mouse donors, each one represented by a column at each time-point.

To determine how the exogenously provided sodium lactate is taken up by CD8+ T cells and incorporated into carbon metabolism, we activated CD8+ T cells in the presence of 11.1 mM [U-^13^C_6_]glucose alone, in combination with 40 mM sodium lactate, or 40 mM [U-^13^C_3_]sodium lactate in combination with 11.1 mM glucose (**Figure 4A** and **Supplementary Figure 4**). Labelling of cellular metabolites was measured by mass spectrometry at 24, 48 and 72 hours after activation (**Figures 4B–D** and **Supplementary Figure 4**). Activation in the presence of [U-^13^C_6_]glucose alone resulted initially (24-48h) in labelling of metabolites in the glycolytic pathway, including phosphoenolpyruvate (PEP), then pyruvate and lactate, followed by TCA cycle intermediates at 48-72 hours. Progressive labelling of ribose, originating from the pentose phosphate pathway (PPP), and the amino acids serine, glycine, alanine, glutamate, proline, and aspartate was also observed, suggesting incorporation of glucose-derived carbons through glycolysis and its branch pathways, as well as the TCA cycle. Addition of sodium lactate significantly displaced the contribution of [U-^13^C_6_]glucose to the labelling of pyruvate and lactate, but not to PEP or ribose (**Figure 4C** and **Supplementary Figure 4**). Glucose labelling of TCA cycle metabolites was markedly decreased by addition of lactate, as was the labelling of amino acids (**Figure 4C** and **Supplementary Figure 4**). Tracing of [U-^13^C_3_]sodium lactate revealed that exogenously sourced lactate enters T cells and is oxidized to pyruvate (**Figure 4D** and **Supplementary Figure 4**). Labelling of metabolites downstream of pyruvate, namely alanine and citrate, occurs rapidly. Within 24 hours, labelling from lactate-derived carbons can be found in the TCA cycle metabolites succinate, fumarate and malate. In addition to alanine, lactate-derived carbons were incorporated into amino acids derived from the TCA cycle such as glutamate, proline and aspartate, but not into amino acids derived from the glycolytic pathway upstream of pyruvate, e.g., serine and glycine (**Figures 4B–D** and **Supplementary Figure 4**). These findings show that exogenous lactate can be taken up, oxidized, and displace glucose as a carbon source for both the TCA cycle and amino acid synthesis in activated CD8+ T cells.

### 3.5 Activation in the presence of lactate modulates the differentiation of CD8+ T cells

**Figure 5:**
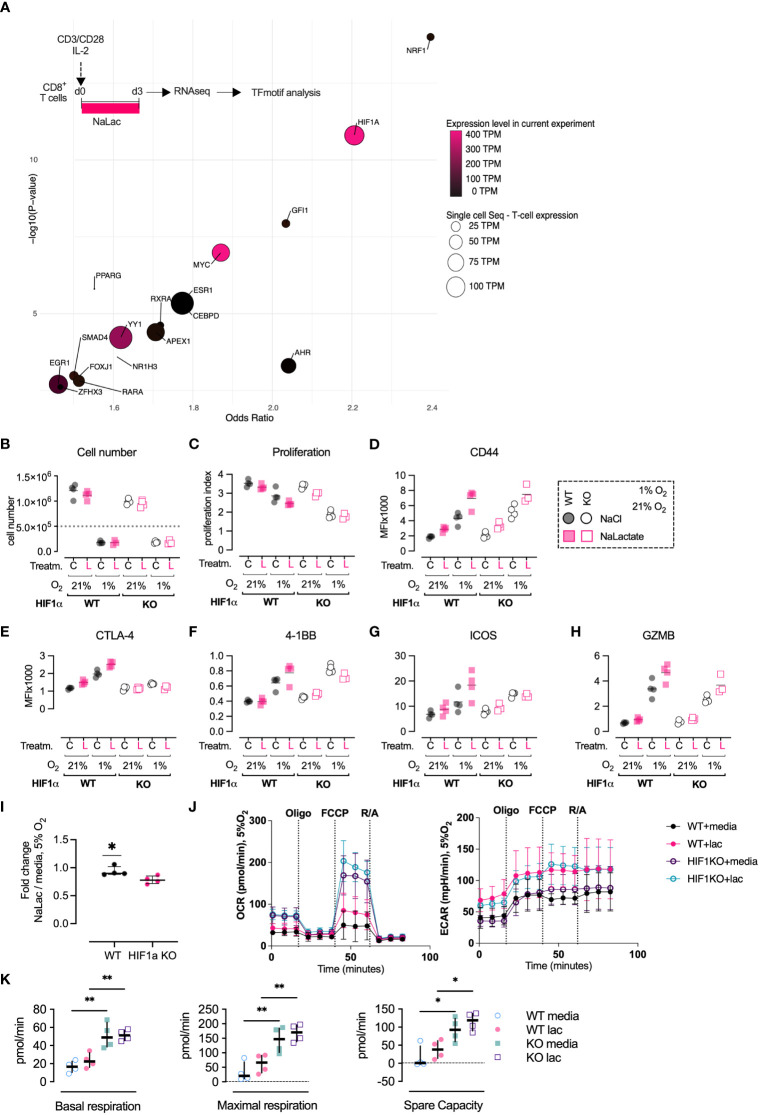
Activation in the presence of lactate modulates the differentiation of CD8+ T cells. **(A)** *In silico* prediction of transcriptional regulators of lactate exposure. Bubble-graph of Transcription factor binding-motif enrichment Odd-ratio (x-axis) and p-value (y-axis) based on transcripts differentially regulated by lactate exposure. Transcript abundance of each transcription factor is denoted by color (present experiment average expression) and size (T cell single-cell average expression). NRF1 was the transcription factor with highest motif enrichment (OR 2.4 p 1e-14) but with low transcript abundance (64 and 14 TPM). HIF-1α had a high motif-enrichment (OR 2.2 p 1.5e-11) and high T-cell transcript abundance (388 and 92 TPM). Data extrapolated from the RNA-seq analysis of CD8+ T cells activated for 72 hours in the presence of 40 mM NaLac or media control. Results were analyzed in silico using Positional weight matrixes of human and rodent transcription factors utilizing enrichR, combined with transcription factor transcript abundance from the present experiemtn and single-cell sequencing data from human T-cells. **(B)** Cell number of wild type (Hif1α^fl/fl^) or HIF-1α deficient CD8+ T cells (Hif1α^fl/fl^ dLck Cre) after 3 days of activation in the presence of either 40 mM NaCl **(C)** or NaLac **(L)**, at 1% or 21% oxygen. Grey dotted line: number of seeded cells. **(C)** Proliferation Index for the assay described in **(B)**. **(D)** Median Fluorescence Intensity (MFI) of surface markers CD44, **(E)** CTLA-4, **(F)** 4-1BB, and **(G)** ICOS, and **(H)** intracellular GZMB levels at three days after activation, as described in **(B)**. **(I)** Fold change of CD8+ T cells numbers in wild type (Hif1α^fl/fl^) versus HIF-1α deficient CD8+ T cells (Hif1α^fl/fl^ dLck Cre) after 3 days of activation in the presence of 40 mM NaLac or media, at 5% oxygen. **(J)** Oxygen consumption rate (OCR) and extracellular acidification rate (ECAR) of wild type (Hif1α^fl/fl^) or HIF-1α deficient CD8+ T cells (Hif1α^fl/fl^ dLck Cre) after 72 hours activation in the presence of 40 mM NaLac or T cell media at 5% O_2_. OCR and ECAR were measured during sequential addition of 1 µM oligomycin (oligo), 1.5 µM FCCP, and 100 nM rotenone combined with 1 µM antimycin A (Rot/Ant). **(K)** Basal respiration, maximal respiration, and spare capacity determined from the assay shown in **(J)**. *p<0.05, **p< 0.005, one-way ANOVA with Tukey’s multiple comparisons test.

Based on the consistent differential expression of critical molecules for metabolism and effector function following lactate exposure during activation of CD8+ T cells, we sought to identify potential up-stream transcriptional regulators that could explain the observed alterations. To this aim, we utilized the 184 genes significantly differentially regulated with lactate exposure, to do a transcription-factor motif-enrichment analysis. Seventeen transcription factors had significant motif-enrichment and were readably detected transcriptionally in T cells. NRF1 was the transcription factor with the highest motif enrichment (OR 2.4 p 1e-14) but with low transcript abundance - 64 TPM (present data) and 14 TPM (single-cell). HIF-1α had a motif-enrichment (OR 2.2 p 1.5e-11) and high T cell transcript abundance - 388 TPM (present data) and 92 TPM (single-cell). The transcription factor with the highest affinity score was HIF-1α (affinity score 4.1, p = 0.002) together with c-MYC (affinity score 4.2, p = 0.002) (**Figure 5A**). The transcription factor analysis indicated that the HIF-1α transcription factor motif is enriched in the genes differentially expressed after exposure to sodium lactate. To follow up on this finding, we measured the differentiation of CD8+ T cells activated in the presence of 40 mM sodium lactate at ambient oxygen tension (21% O_2_) or hypoxic conditions (1% O_2_), using wild type or HIF-1α null CD8+ T cells (**Figures 5B**). Activation in hypoxic conditions severely limited CD8+ T cell proliferation but led to greater expression of the effector markers CD44, CTLA-4, 4-1BB, GZMB and ICOS, in agreement with previously published data (**Figures 5C–H**) (4, 49). Exposure to lactate led to an increase in expression of effector markers, which was further enhanced when combined with hypoxia (**Figures 5B–H**). Previous findings showed that effector differentiation was compromised in the absence of HIF-1α (4, 49, 50). Here, we observed that CD8+ T cells lacking HIF-1α failed to upregulate CTLA-4 in all of the tested culture conditions, suggesting a high level of HIF-1α-dependency (**Figure 5E**). In contrast, expression of CD44, 4-1BB, GZMB and ICOS was increased in hypoxia even in the absence of HIF-1α. The additive effect of lactate and hypoxia on the expression of 4-1BB and ICOS was lost in HIF-1α null CD8+ T cells. This HIF-dependency was not observed for GZMB and CD44. To gain insights on the role of HIF-1α in regulating the metabolism of CD8+ T cells, especially in the presence of lactate, we activated murine CD8+ T cells under mild hypoxic conditions (5% O2) and with or without sodium lactate. Interestingly, the lack of HIF1α led to increased basal and maximal oxidative respiration as well as spare oxidative capacity, independently of lactate presence and even at reduced oxygen tension (**Figures 5I–K**). This confirms the critical role played by HIF1α in triggering glycolysis. The addition of lactate leads to elevated extracellular acidification rate (ECAR) in both WT and HIF1α KO cells, suggesting saturation of glycolytic activity. This is in accordance with the increased oxygen consumption rate. Altogether, these results show that lactate and hypoxia have additive effects in driving expression of CD8+ T cell effector markers and metabolic activity and indicate that this relationship is in part dependent on HIF-1α.

## 4 Discussion

Here we show that the lactate anion - even at doses in the higher physiological range - is well tolerated by CD8+ T cells and can be taken up and incorporated into T cell metabolism, replacing glucose as the preferred carbon source for biomolecules downstream pyruvate. Moreover, by shaping the transcriptome, lactate can ultimately alter the fate of CD8+ T cells.

In line with our previous findings (30), increased lactate availability during activation enhanced the expression of cytotoxic molecules such as Granzyme B and Interferon-γ. The enhanced expression of effector molecules was paired with increases in CD44, co-activators 4-1BB and ICOS, and co-inhibitor CTLA-4 and a reduction in lymph node homing marker CD62L. Lactate also reduced the levels of a master regulator of CD8+ T cell differentiation, the transcription factor T-bet. The change in expression of functional molecules was accompanied by a coordinated upregulation of genes from the TCA cycle, indicating substantial effects on T cell metabolism.

While the metabolic consequence of acute exposure to external sodium lactate was repression of the glycolytic flux and a concurrent boosting of mitochondrial respiration, the reduced acidification rate was only temporary and dependent on lactate concentration remaining high. When lactate was removed, such as at the end of the prolonged exposure over the activation period in our settings, the cellular energetic flux was re-established and at a higher pace. In fact, as shown by our data, at day 3 after activation lactate-treated CD8+ T cells showed an increased glycolytic as well as oxidative rate. This underlines the energetic role of lactate, which does not impair, but rather rewires the metabolic machinery of the activated cell. In other words, exposure to high doses of lactate, in absence of pH-lowering conditions, seems to be sufficient to reprogram T cell metabolism. This is in agreement with the growing evidence that the microenvironmental composition can shape T cell metabolism and function. (11, 31, 51, 52).

Transport of L-lactate into T cells was first described in the early 1990s (53), and its uptake was shown to be dependent on the activation state of the T cells. Activating CD8+ T cells with carbon-labeled lactate allowed us to trace its active uptake and incorporation into carbon metabolism. After being oxidized to pyruvate, exogenous lactate was not incorporated into upstream metabolites in the glycolytic cascade. Instead, lactate-derived carbons were specifically incorporated into downstream TCA cycle intermediates and pyruvate-derived amino acids such as alanine. Interestingly, this resulted in the displacement of glucose as the preferred source of carbons for TCA intermediates. It has previously been shown in the systemic setting that lactate can be a primary source of carbon for the TCA cycle and thus of energy, with a turnover rate exceeding that of glucose (18). Thus, our data indicate that exogenous lactate, at least when present in high doses, replaces glucose as the preferred substrate to generate specific amino acids and TCA cycle intermediates in activated CD8+ T cells. Unexpectedly, the presence of lactate seems to indirectly impair how glucose feeds the serine, glycine, one-carbon (SGOC) metabolism, although no contribution of lactate-derived carbons could be detected in either glycine or serine. This suggests that lactate, through the higher metabolic turnover, can contribute to enhanced uptake of extracellular amino acids. Recently, serine uptake has been shown essential for *de novo* nucleotide biosynthesis in CD8+ T cells during proliferation (54). Hence, in addition to enhancing the metabolic rate and altering the intracellular route of glucose, lactate may modulate cellular metabolism to support bioenergetics, enhancing the overall immunological activity of CD8+ T cells.

Transcription factor motif analysis based on the differentially expressed genes after activation in the presence of L-lactate indicated involvement of several transcription factors. In particular, a strong enrichment of the transcription factor HIF-1α was observed. Previous work has shown that HIF-1α is necessary for effector differentiation of CD8+ T cells (49) and contributes to the metabolic switch to aerobic glycolysis occurring early during activation (3) and it therefore emerged as a candidate for mediating the effect of lactate.

Activating CD8+ T cells under hypoxic conditions showed that lactate and hypoxia have additive effects on effector markers. While lactate enhanced expression of ICOS and 41BB, in HIF-1α null CD8+ T cells this effect was lost, suggesting a role of HIF-1α in mediating the effects of lactate. However, as indicated from the transcription factor analysis where enrichment was found for several key transcription factors, the shaping of CD8+ T cell fate by lactate was only partially dependent on HIF-1α.

The use of distally produced endogenous lactate as a fuel for oxidative respiration has been previously reported in other cell types, both malignant and non-malignant (16, 18). The capacity of lactate to move freely between cells, tissues, and organs, be taken up through the monocarboxylate transporters and to be converted into useful substrates for metabolism through the lactate dehydrogenase enzymes and thereby shifting the metabolism of its target cells, suggests lactate to convey metabolic messages on a cellular as well as systemic level. Here we showed that extracellular lactate was used as a preferred source of carbon by CD8+ T cells during the early effector differentiation phase. In addition, lactate altered the metabolic flux of the cells and shaped effector differentiation. Altered lactate availability arises not only during major physiological or pathological conditions such as high intensity exercise or sepsis. Locally-produced lactate originating from primed T cells might, for example, function as a quorum-sensing mechanism to cross-potentiate the early T cell response.

The exceptional plasticity of T cells represents one of their most distinctive features, and the ability to rapidly adapt to diverse environmental conditions, including reduced oxygen availability and nutrient deprivation, is of crucial importance for their immune activity. Here we show that lactate can act as alternative source of energy as well as immunomodulatory molecule in CD8+ T cells. Further research is warranted to understand whether incorporation of lactate into carbohydrate metabolism is required for its shaping of effector functions.

## Data availability statement

All data generated are included in the manuscript and supporting files. The RNA sequencing data discussed in this publication have been deposited in NCBI's Gene Expression Omnibus (55) and are accessible through GEO Series accession number GSE190808 (https://www.ncbi.nlm.nih.gov/geo/query/acc.cgi?acc=GSE190808).

## Ethics statement

Ethical review and approval was not required for the study on human participants in accordance with the local legislation and institutional requirements. Written informed consent for participation was not required for this study in accordance with the national legislation and the institutional requirements. All experiments and protocols were approved by the regional animal ethics committee of Northern Stockholm (dnr N78/15, N101/16).

## Author contributions

LB and PV contributed equally toward experimental design, performance, and analysis; interpretation of results; drafting and revision of the manuscript. PG contributed to the design, performance, and analysis of carbon tracing experiments. PC conducted experiments. IF contributed to experiment design and discussion. DB contributed to the bioinformatic analysis of RNA-seq data and GEO submission. ER contributed to the transcription factor motif enrichment analysis. HR contributed to the study conceptualization. RJ and HR supervised the work and revised the manuscript. All authors discussed and edited the manuscript. All authors contributed to the article and approved the submitted version.

## Supplementary material

Supplementary Figure 1 Sodium lactate, but not lactic acid, is well-tolerated by CD8+ T cells. **(A)** Osmolality of increasing doses of sodium chloride (NaCl) and sodium lactate (NaLac) solutions in complete RPMI. Horizontal dotted line: RPMI reference. Vertical dotted line: osmolality of 40 mM NaCl or NaLac solution in RPMI. **(B)** pH at room temperature of NaCl, NaLac, and Lac acid solutions in complete RPMI. Horizontal blue line: media reference. **(C)** Mouse CD8+ T cells were activated for 72 hours in the presence of 40 mM NaCl or NaLac. At the end of the activation period, cells were washed and absolute cell number and proliferation index at each cell generation were determined by CFSE dilution. Mean and SEM of n = 3 independent mouse donors. *P<0.001 repeated-measures two-way ANOVA with Sidak’s multiple comparison test. **(D)** Effect of varying concentrations of NaCl or NaLac on human CD8+ T cell number, 96 hours after activation with anti-CD3/CD28 beads and in the presence of IL-2. Dotted line and grey range: average of non-activated (naïve) cells. Solid light blue line: cells activated in the presence of plain culture media (control). N = 2 independent human donors. **(E)** Human CD8+ T cells purified from healthy donor PMBCs were activated in the presence of 40 mM NaCl or NaLac for 4 days. Long-term expansion was continued after NaCl/NaLac removal at day 4, with addition of fresh media and IL-2. Cell number was measured at day 4, 7 and 14. Data are the median and interquartile range of n = 10 human donors. **(F)** Mouse CD8+ T cells were activated for 72 hours in the presence of either 40 mM NaCl (black line) or 40 mM NaLac (pink line). Flow cytometry histograms showing intracellular Granzyme B (GZMB) levels at day 3 after activation and at each cell division (determined by CTV peaks). **(G)** Median Fluorescence Intensity (MFI) of GZMB levels at each cell division of mouse CD8+ T cells activated as described in **(F)**. Lines represent the median of n = 6 biological replicates. ****P<0.0001 Two-way Anova with Šídák’s multiple comparisons test. **(H)** Heatmap and clustering of genes from RNA-seq experiment described in . Only genes that were found significantly deregulated in any of the comparisons are shown. Each gene (row) is standardized (z) to a mean of 0 and standard deviation (SD) of 1. “Treatment” indicates activation in the presence of either media (light blue) or NaLac (pink), or non-activated cells (naive, grey). The “weight” heatmap indicates the degree of influence of each sample on the overall analysis. Data is provided as **Supplementary Figure 1**-source data 1 (panel H). **(I)** Median Fluorescence Intensity of CD62L, CD127, CD25, PD-1, CTLA-4, LAG-3, 4-1BB, T-bet, and CD27 at each cell division, 72 hours after activation with 40 mM NaCl (black line) or NaLac (pink line). Significance was calculated using RM two-way ANOVA with the Geisser-Greenhouse correction and Sidak’s multiple comparison test.

Supplementary Figure 2 Lactate modulates CD8+ T cell metabolism. **(A)** Heatmaps generated from RNA-seq data, focusing on the differentially expressed TCA cycle (left) or glycolytic (right) genes, in NaLac- relative to media-treated CD8+ T cells. Colors indicate the degree of differential expression (as Log2 of the fold change) of genes in each pathway. Significant genes (adjPval ≤ 0.01) are indicated in red. Data is provided as **Supplementary Figure 2**-source data 1 panel **(A)**. **(B)** Experimental design. Human CD8+ T cells were isolated from the blood of healthy donors and activated for 12 hours in IL-2 and. After 12 hours, cells were transferred in a seahorse XF analyzer for metabolic flux analysis after injection of 40 mM NaCl or sodium lactate (NaLac). **(C)** Glycolytic stress test of human CD8+ T cells 12 hours after activation as in **(B)**. Graphs show extracellular acidification rate (ECAR) and oxygen consumption rate (OCR) during sequential injection of either media, or 40 mM NaCl, or 40 mM NaLac, followed by 10 mM glucose, 1 µM oligomycin (oligo) and 50 mM 2-deoxyglucose (2-DG). ECAR and OCR are normalized to baseline levels. Data is the mean and SEM of 4 technical replicates of one representative donor. **(D)** Basal glycolysis, basal mitochondrial respiration, and glycolytic capacity determined from the assay shown in **(C)**. Lines indicate the median of n = 4 technical replicates of one representative donor. Dotted line: median of NaCl-treated cells. **P<0.01 one-way ANOVA with Holm-Šídák’s multiple comparisons test between each donor’s technical replicates. **(E)** Mouse CD8+ T cells were activated for 72h with anti-CD3/CD28 beads or kept in a naïve-like state (non-activated) with IL-7 over the same period. After activation, cells were analyzed in a Seahorse XF96 Flux Analyzer to test their glycolytic function, *via* consecutive injection of 10 mM glucose (glc), 1 μM oligomycin (oligo) and 50 mM 2-deoxyglucose (2-DG). ECAR: Extracellular Acidification Rate. Data are the median and interquartile range of n = 4 independent mouse donors. **p<0.005 one-way ANOVA with Tukey’s multiple comparisons test. **(F)** Extracellular lactate concentration from the supernatant of mouse CD8+ T cells, 72 hours after *in vitro* activation. Lactate levels were measured by enzymatic assay. Dotted line: non-activated (naïve) cells. N = 3 individual mice. **P<0.01 paired t test, two-tailed. **(G)** 15 µg of mouse CD8+ T cell protein cytoplasmic extracts from 0, 6, 12, 24, 48, and 72 hours post-activation were probed with antibodies against MCT1, MCT4, and GZMB. Total protein stain (TPS) was used as loading control. Data is provided as **Supplementary Figure 2**-source data 2 panel **(G)**. **(H)** Protein quantification from experiment shown in **(G)** (normalized to total protein stain, TPS) and mRNA (normalized to expression of the housekeeping gene *Hprt*) levels of MCT1, MCT4 and GZMB over time since activation. **(I)** Mouse CD8+ T cells were activated for 72 hours in the presence of the monocarboxylate transporter-1 (MCT1) inhibitor AZD3985 in combination with 40 mM NaLac or culture media. A non-linear fit ([Inhibitor] vs. response – Variable slope (four parameters)) was used to determine IC_50_. Blue area = range of untreated controls; dotted line: non-activated (naive) cells. **(J)** Estimated protein mean copy number of Ldha and Ldhb at day 0, 1, and 4 after activation of mouse P14 transgenic CD8+ T cells in the presence of IL-2. Data were obtained from the public ImmPRes database (http://immpres.co.uk/) (Howden et al., 2019). Lines represent the mean and standard deviation. **(K)** CD8+ T cells were activated for 72 hours in the presence of increasing doses of the LDH inhibitor GSK2837808A and either 40 mM NaCl or NaLac. Cell numbers are expressed as fold change relative to seeding cell number.

Supplementary Figure 3 Early exposure to lactate alters CD8+ T cell metabolism and gene expression. Same data as in , shown as heatmaps of the differentially expressed TCA cycle (left) or glycolytic (right) genes, in NaLac- relative to media-treated CD8+ T cells. Colors indicate the degree of differential expression (as Log2 of the fold change) of genes in each pathway. Significant genes (adjPval ≤ 0.01) are indicated in red. Data are provided as **Supplementary Figure 3**-source data 1.

Supplementary Figure 4 Lactate is incorporated in cellular metabolism and displaces glucose. Same experiment as in but shown as metabolic diagrams of glucose- or lactate-derived carbons incorporation into cellular metabolites at 24, 48, and 72 hours after activation. Top: Experimental design and schematic representation of the labelled molecules. Bottom: lines showing the fraction of each metabolite labelled by [U-^13^C_6_]glucose alone (black), or in the presence of sodium lactate (pink), or by [U-^13^C_6_]sodium lactate (orange). Lines connect the median of n = 3 independent mice donors. *P<0.05, **P<0.01, ***P<0.001, repeated-measures two-way ANOVA with Sidak’s multiple comparison test comparing glucose alone and glucose with addition of 40 mM NaLac. Data provided as **Supplementary Figure 4**-source data 1.
