# Supplementary figures and images for: Lactate exposure shapes the metabolic and transcriptomic profile of CD8+ T cells
Source: Front Immunol. 2023 Feb 27;14:1101433. doi: 10.3389/fimmu.2023.1101433 (PMC10008868; doi:10.3389/fimmu.2023.1101433)

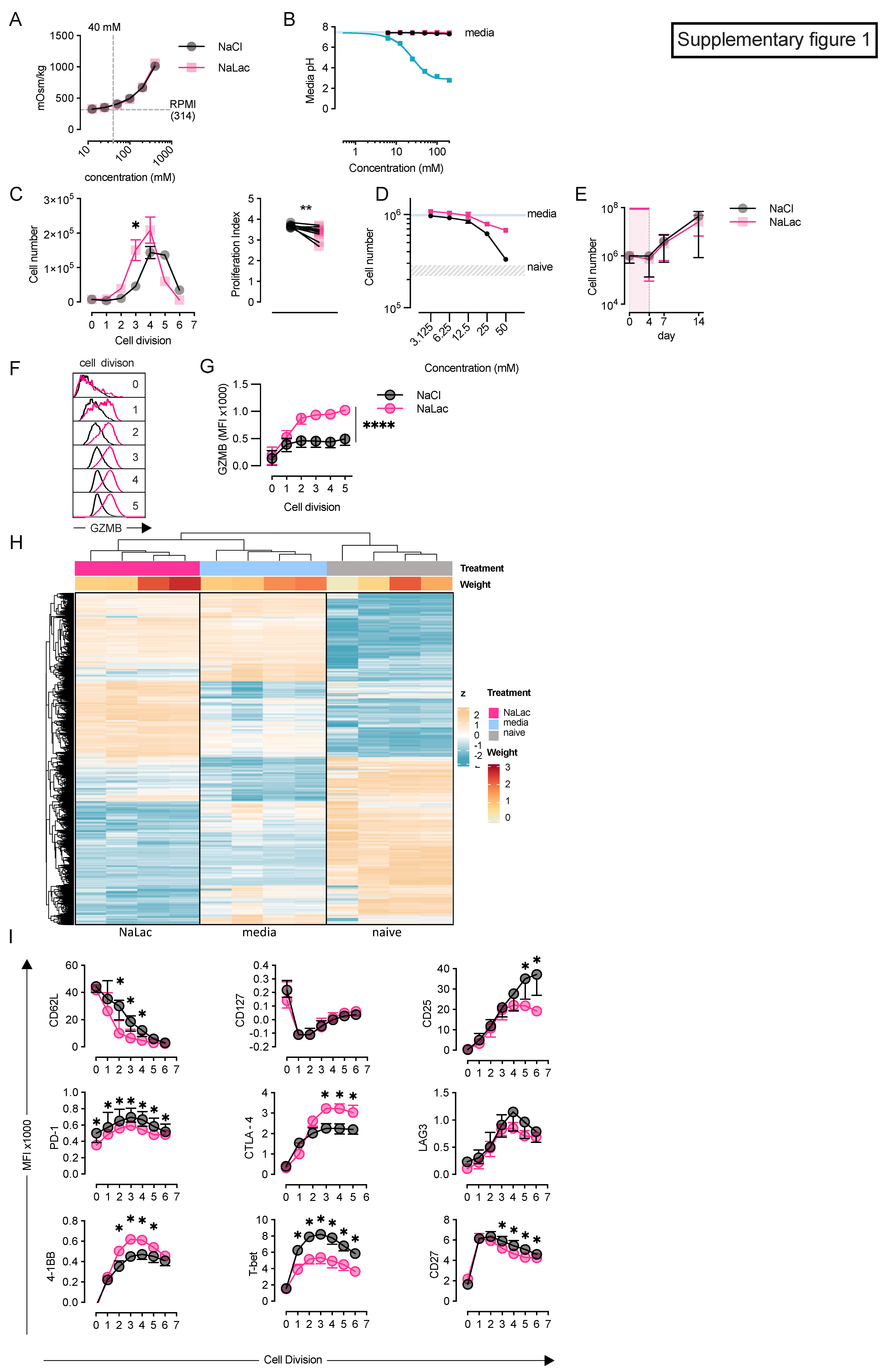

Supplement: Supplementary Figure 1 — Sodium lactate, but not lactic acid, is well-tolerated by CD8+ T cells. (A) Osmolality of increasing doses of sodium chloride (NaCl) and sodium lactate (NaLac) solutions in complete RPMI. Horizontal dotted line: RPMI reference. Vertical dotted line: osmolality of 40 mM NaCl or NaLac solution in RPMI. (B) pH at room temperature of NaCl, NaLac, and Lac acid solutions in complete RPMI. Horizontal blue line: media reference. (C) Mouse CD8+ T cells were activated for 72 hours in the presence of 40 mM NaCl or NaLac. At the end of the activation period, cells were washed and absolute cell number and proliferation index at each cell generation were determined by CFSE dilution. Mean and SEM of n = 3 independent mouse donors. *P<0.001 repeated-measures two-way ANOVA with Sidak’s multiple comparison test. (D) Effect of varying concentrations of NaCl or NaLac on human CD8+ T cell number, 96 hours after activation with anti-CD3/CD28 beads and in the presence of IL-2. Dotted line and grey range: average of non-activated (naïve) cells. Solid light blue line: cells activated in the presence of plain culture media (control). N = 2 independent human donors. (E) Human CD8+ T cells purified from healthy donor PMBCs were activated in the presence of 40 mM NaCl or NaLac for 4 days. Long-term expansion was continued after NaCl/NaLac removal at day 4, with addition of fresh media and IL-2. Cell number was measured at day 4, 7 and 14. Data are the median and interquartile range of n = 10 human donors. (F) Mouse CD8+ T cells were activated for 72 hours in the presence of either 40 mM NaCl (black line) or 40 mM NaLac (pink line). Flow cytometry histograms showing intracellular Granzyme B (GZMB) levels at day 3 after activation and at each cell division (determined by CTV peaks). (G) Median Fluorescence Intensity (MFI) of GZMB levels at each cell division of mouse CD8+ T cells activated as described in (F). Lines represent the median of n = 6 biological replicates. ****P<0.0001 Two-way Anova w [file Image_1.tiff]

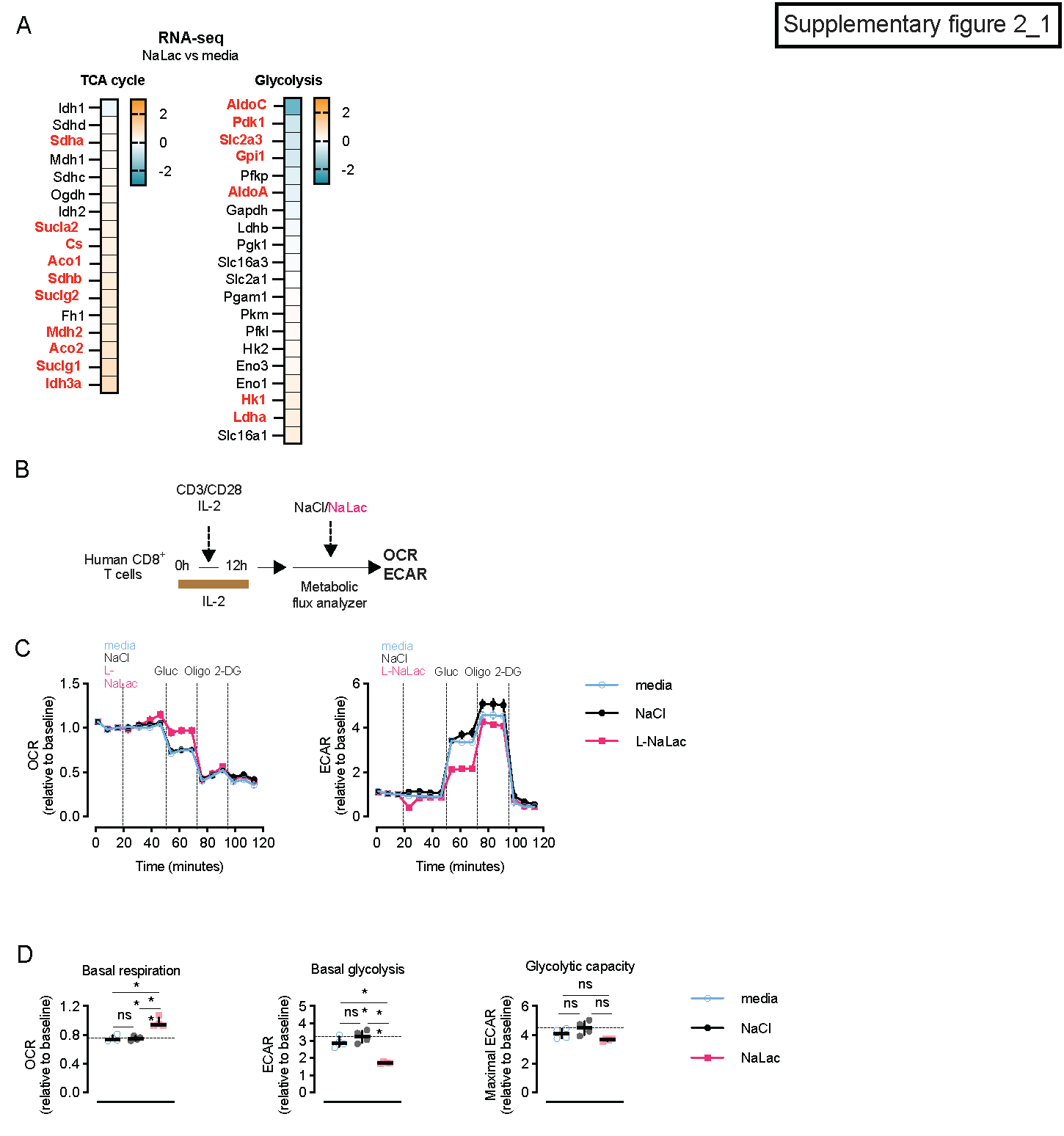

Supplement: Supplementary Figure 2 — Lactate modulates CD8+ T cell metabolism. (A) Heatmaps generated from RNA-seq data, focusing on the differentially expressed TCA cycle (left) or glycolytic (right) genes, in NaLac- relative to media-treated CD8+ T cells. Colors indicate the degree of differential expression (as Log2 of the fold change) of genes in each pathway. Significant genes (adjPval ≤ 0.01) are indicated in red. Data is provided as Supplementary Figure 2 -source data 1 panel (A). (B) Experimental design. Human CD8+ T cells were isolated from the blood of healthy donors and activated for 12 hours in IL-2 and. After 12 hours, cells were transferred in a seahorse XF analyzer for metabolic flux analysis after injection of 40 mM NaCl or sodium lactate (NaLac). (C) Glycolytic stress test of human CD8+ T cells 12 hours after activation as in (B). Graphs show extracellular acidification rate (ECAR) and oxygen consumption rate (OCR) during sequential injection of either media, or 40 mM NaCl, or 40 mM NaLac, followed by 10 mM glucose, 1 µM oligomycin (oligo) and 50 mM 2-deoxyglucose (2-DG). ECAR and OCR are normalized to baseline levels. Data is the mean and SEM of 4 technical replicates of one representative donor. (D) Basal glycolysis, basal mitochondrial respiration, and glycolytic capacity determined from the assay shown in (C). Lines indicate the median of n = 4 technical replicates of one representative donor. Dotted line: median of NaCl-treated cells. **P<0.01 one-way ANOVA with Holm-Šídák’s multiple comparisons test between each donor’s technical replicates. (E) Mouse CD8+ T cells were activated for 72h with anti-CD3/CD28 beads or kept in a naïve-like state (non-activated) with IL-7 over the same period. After activation, cells were analyzed in a Seahorse XF96 Flux Analyzer to test their glycolytic function, via consecutive injection of 10 mM glucose (glc), 1 μM oligomycin (oligo) and 50 mM 2-deoxyglucose (2-DG). ECAR: Extracellular Acidification Rate. Data are the median and interquartile range [file Image_2.tiff]

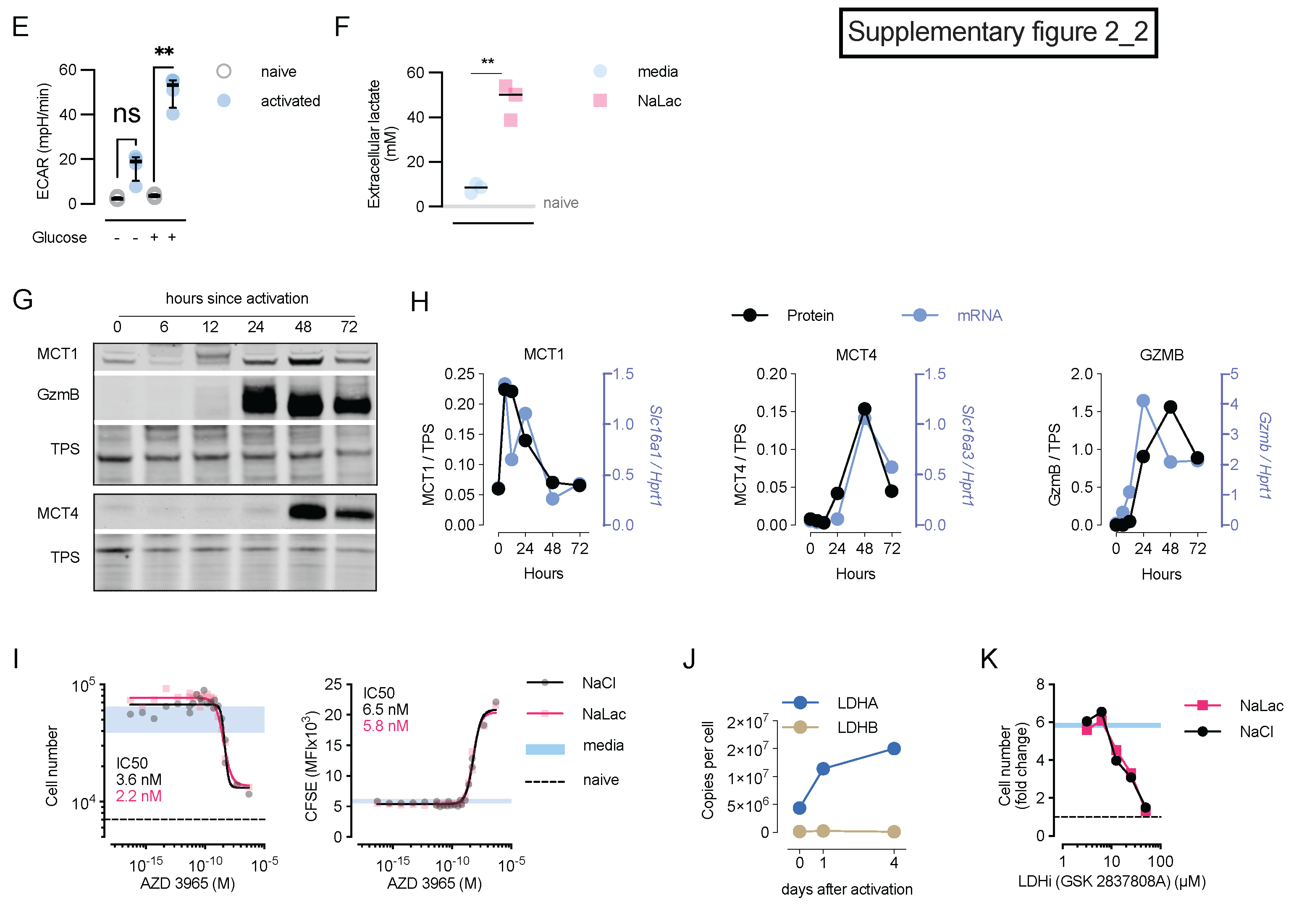

Supplement: Supplementary Figure 3 — Early exposure to lactate alters CD8+ T cell metabolism and gene expression. Same data as in , shown as heatmaps of the differentially expressed TCA cycle (left) or glycolytic (right) genes, in NaLac- relative to media-treated CD8+ T cells. Colors indicate the degree of differential expression (as Log2 of the fold change) of genes in each pathway. Significant genes (adjPval ≤ 0.01) are indicated in red. Data are provided as Supplementary Figure 3 -source data 1. [file Image_3.tiff]

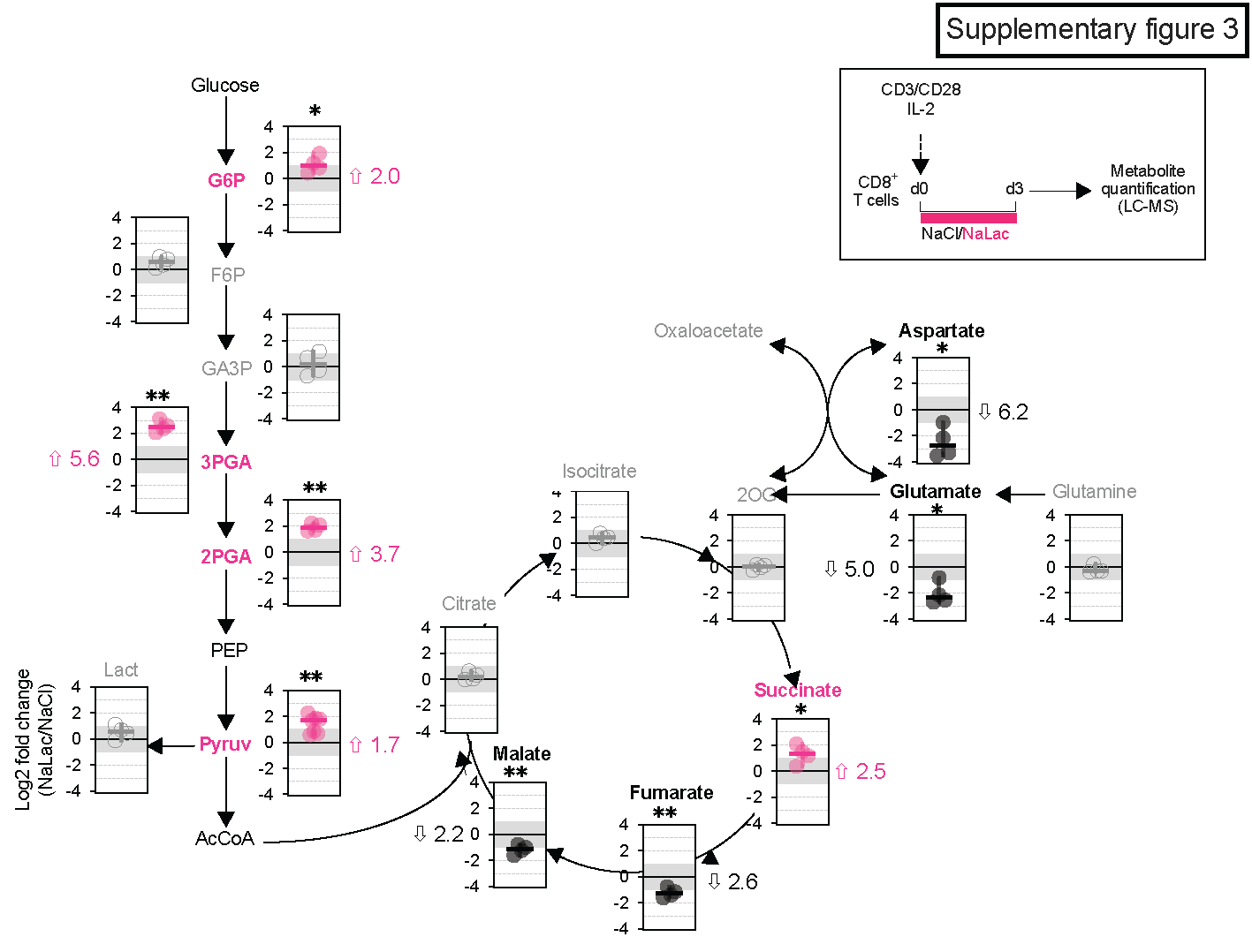

Supplement: Supplementary Figure 4 — Lactate is incorporated in cellular metabolism and displaces glucose. Same experiment as in but shown as metabolic diagrams of glucose- or lactate-derived carbons incorporation into cellular metabolites at 24, 48, and 72 hours after activation. Top: Experimental design and schematic representation of the labelled molecules. Bottom: lines showing the fraction of each metabolite labelled by [U-13C6]glucose alone (black), or in the presence of sodium lactate (pink), or by [U-13C6]sodium lactate (orange). Lines connect the median of n = 3 independent mice donors. *P<0.05, **P<0.01, ***P<0.001, repeated-measures two-way ANOVA with Sidak’s multiple comparison test comparing glucose alone and glucose with addition of 40 mM NaLac. Data provided as Supplementary Figure 4 -source data 1. [file Image_4.tiff]

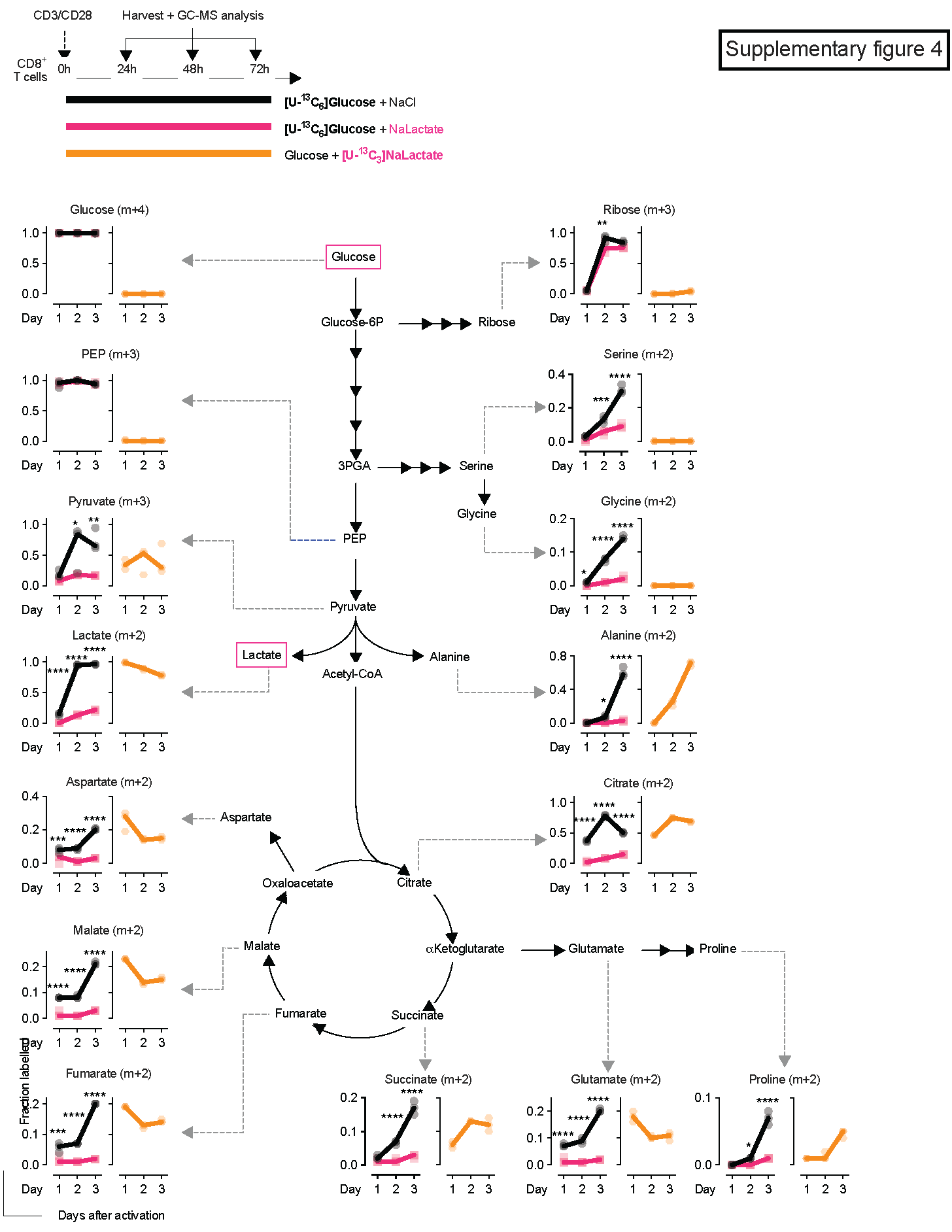

Supplement: Supplementary file 5 [file Image_5.tiff]
